# Compulsive Sexual Behavior Online and Non-online in Adult Male Patients and Healthy Controls: Comparison in Sociodemographic, Clinical, and Personality Variables

**DOI:** 10.3389/fpsyt.2022.839788

**Published:** 2022-05-03

**Authors:** Vega González-Bueso, Juan José Santamaría, Oriol Caro-Pérez, Daniel Fernández, Marta Baño-Alcazar, Susana Jiménez-Murcia, Anders Håkansson, Amparo del Pino-Gutiérrez, Joan Ribas

**Affiliations:** ^1^Atención e Investigación en Socioadicciones (AIS), Mental Health and Addictions Network, Generalitat de Catalunya, Sistema Sanitari Integral d’Utilització Pública de Catalunya (SISCAT), Barcelona, Spain; ^2^CELBIOTECH_Paper Engineering Research Group, Universitat Politècnica de Catalunya (UPC), Barcelona, Spain; ^3^Department of Statistics and Operations Research (DEIO), Universitat Politècnica de Catalunya BarcelonaTec (UPC), Barcelona, Spain; ^4^Institute of Mathematics of UPC – BarcelonaTech (IMTech), Barcelona, Spain; ^5^Instituto de Salud Carlos III, Centro de Investigación Biomédica en Red de Salud Mental (CIBERSAM), Madrid, Spain; ^6^Pathological Gambling Unit, Department of Psychiatry, Bellvitge University Hospital-IDIBELL, Barcelona, Spain; ^7^Ciber Fisiopatología Obesidad y Nutrición (CIBERObn), Instituto de Salud Carlos III, Madrid, Spain; ^8^Department of Clinical Sciences, School of Medicine and Health Sciences, University of Barcelona, Barcelona, Spain; ^9^Division of Psychiatry, Department of Clinical Sciences Lund, Faculty of Medicine, Lund University, Lund, Sweden; ^10^Malmö Addiction Center, Competence Center Addiction, Region Skåne, Malmö, Sweden; ^11^Department of Public Health, Mental Health and Maternal and Child Health Nursing, Nursing School, University of Barcelona, Barcelona, Spain

**Keywords:** compulsive sexual behavior, personality, psychopathology, profiles, behavioral addictions

## Abstract

**Background and Aims:**

Compulsive sexual behavior (CSB) is characterized by a persistent pattern of failure to control sexual impulses, resulting in repetitive sexual behavior over a prolonged period that causes marked discomfort in personal, family, social, school, work or in other functional areas. The evolution of the worldwide incidence of this disorder warrants further studies focused on examining the characteristics of the affected people. The purpose of this study was to compare online compulsive sexual behavior (when the problematic sexual practices were online) and non-online compulsive sexual behavior (when the problematic sexual practices were in-person) patients (OCSB and non-OCSB, respectively), and healthy controls in terms of sexual behavior, sociodemographic variables and psychopathology and personality characteristics.

**Method:**

A sample of 80 CSB male patients consecutively admitted to our Behavioral Addictions Unit and 25 healthy male controls, participated in the study. The CSB group was comprised by 36 online CSB patients (mean age 42.25, SD: 10.0) and 44 non-online CSB patients (mean age 43.5, SD: 11.9). Scores on the Sexual Compulsivity Scale, Temperament and Character Inventory-Revised, Symptom CheckList-90 Items-Revised, State-Trait Anxiety Index, and additional demographic, clinical, and social/family variables related to sexual behaviors between the three groups were compared.

**Results:**

When compared with healthy controls, both clinical groups showed higher psychopathology in all measures as well as higher harm avoidance and self-transcendence and lower self-directness and cooperativeness. When comparing OCSB and non-OCSB patients, results showed that non-OCSB patients exhibited higher prevalence of sexually transmitted diseases, higher percentage of homosexual and bisexual orientation and higher scores in anxiety and in sexual impulse control failure.

**Conclusion:**

Both online and non-online CSB patients may experience a variety of comorbid psychological and medical problems. Patients with non-OCSB may suffer more consequences that are negative. Therefore, these results should be considered when designing the most convenient therapeutic approach. Whether sexual orientation plays a role in treatment needs and treatment response in CSB, should be further explored in future studies.

## Introduction

The Diagnostic and Statistical Manual (DSM-5) (1) introduced a new sub-category named “Non-substance-related disorders” included in the category “Substance-Related and Addictive Disorders.” This sub-category includes an addictive disorder not involving substance use, the Gambling Disorder. Apart from the inclusion of the “Gambling Disorder” (F63.0), the DSM-5 committee members considered other conditions such as Internet-gaming disorder (2) or hypersexuality disorder (3, 4). Finally, although several DSM-5 working groups (5) proposed to include the Hypersexual Disorder in the manual. Its inclusion was rejected, and nor was this nosological entity incorporated in the Section III (reserved for conditions that require further study), due to multiple reasons, including the lack of data in important areas (6).

Hypersexuality has been described as a non-controlled pattern of recurrent, intense, and excessive preoccupation with sexual behavior, urges and fantasies that causes adverse consequences and significant psychological distress (5, 7). However, the research suggests the theoretical conceptualizations of hypersexuality, are unlikely to provide a complete description to the diverse presentations and experiences of the condition, and that hypersexuality is diverse, complex and most likely relate to a range of risk factors present across individuals (8).

Similarly, compulsive sexual behavior (CSB) was defined classically as repetitive behaviors mediated by the behavioral attempts to reduce anxiety and other dysphoric affects (e.g., shame and depression) with an underlying obsessive component (9). Despite of the active scientific discussion about whether disorder constitutes a behavioral addiction (10), it has not been included in the International Classification of Diseases 11th (ICD-11) addictive disorders category but in the Impulse Control Disorder category (11). In this classification, the problem is defined as a persistent pattern of failure to control sexual impulses, resulting in repetitive sexual behavior over a prolonged period (6 months or more) that causes marked discomfort in personal, family, social, school, work, or in other functional areas (12).

Mental health professionals recognize the problematic sexual behavior as a problem with clinical relevance. The increase in this demand is congruent with the increased awareness shown by different groups such as health workers, politicians, educators, and researchers. There is a lack of studies involving large samples and the real prevalence of CSB remains unclear. However, according to the Society for the Advancement of Sexual Health (2012), the prevalence of CSB among the general United States population is between 3 and 6%. In line with this, some studies estimate rates of 3–6% (5), mainly affecting adult males (80% or more) (13). Moreover, there is growing evidence of the potentially serious consequences of not treating this condition (14), including emotional and relational problems and risky sexual behaviors leading to sexually transmitted infections such as HIV/AIDS, as well as physical injuries (15, 16). Among male treatment seekers, the most reported clinical associated behaviors are pornography use, compulsive masturbation, various sexual partners, casual/anonymous sex encounters with strangers, and prostitution consumption (13, 17, 18).

Since the popularization of the Internet, new forms of sexual behaviors have emerged (10). This technology has allowed the practice or consumption of sex in new ways (such as online pornography, online sexual chatting, and sexting), causing problems among a small but significant part of the population. Consequences of the problematic online sexual practices are similar to the in-person form and include risky sexual behaviors (19), professional and financial problems (20), interpersonal isolation (21), and online compulsive sexual behavior (OCSB) (22, 23). Less clear is the relationship between problematic online sexual practices and offline sexual difficulties (such as erectile disorder, premature ejaculation, excitation disorder, sexual pain disorder, or orgasmic disorder), some reviews finding that the use of online sexual content can condition sexual arousal causing difficulties (24), while others showing contradictory data and no causal relationship (25).

Although several studies have explored CSB (26–28), the correlates among OCSB symptoms and sociodemographic, psychopathology, and personality variables has rarely been investigated in clinical groups. The few studies analyzing this question have found that, with respect to demographics, CSB is associated with being male (29). Despite these results in clinical population, it is not clear if CSB is gender–specific, the majority of research in general population is based on studies with male samples, and little studies has been performed on CSB in women (30–32). When gender-representative studies have been done in general population, that gender differences are less pronounced (2–3 men with CSB to every 1 woman ratio).

Regarding psychopathology, although studies focusing on CSB or “sex addiction” do not consider whether the behavior is in-person or online, results show comorbidity with anxiety and depression (33, 34), and with other addiction problems, including substance abuse or problem gambling (13, 35). Post-traumatic stress and traumatic episodes also seem to be related to excessive sexual behavior (35). Finally, with respect to personality traits associated to CSB, some authors have related the disorder with high novelty seeking and low self-directedness (36). However, the personality factors involved in its development and maintenance remain scarcely explored.

Taking into account the existing literature with respect to CSB, we can conclude that most of the previous studies did not separate in-person from online behavior. Moreover, most of the previous data have been conducted in non-clinical settings and is based on surveys. Given the scarcity of research performed in clinical samples, the purpose of this study was to identify socio-demographic, sexual behaviors, sexual problem severity, psychopathology, and personality characteristics associated with CSB and OCSB patients from a clinical setting, as well as to compare them with a healthy control group. These analyses can help in the conceptualization of patients consulting for CSB, and these comparisons among individuals can contribute to clarifying some of the results found in previous research regarding clinical profiles, and can help to improve the clinical treatments.

## Materials and Methods

### Participants

The present study was conducted between June 2018–March 2019 and January 2021–March 2021. The initial sample included 87 patients with CSB who were consecutive referrals for assessment and outpatient treatment at the Behavioral Addiction Unit in the mental health center AIS-PRO JUVENTUD (Care and Research in Behavioral Addiction) (AIS), located in Barcelona, Spain. The final sample included 80 participants: 36 OCSB and 44 non-online CSB. A patient was classified as OCSB when there was no face-to-face contact with other people in the uncontrolled sexual behavior. From the initial sample, seven individuals were excluded because they had both OCSB and non-OCSB. However, when compared to the final sample, they did not show greater severity of the disorder measured by SCS (37).

The control group included 25 healthy persons of similar age, was recruited by convenience (verbal approaching) at the same area (Barcelona); they were asked to volunteer and recruited as healthy controls after signing an informed consent form.

The sample size calculation was based on the standard deviations of the questionnaire of the SCS (38). Setting an alpha risk of 0.05 and a beta risk of 0.20 in a two-sided test with a 10% estimated dropout rate, the minimum sample size in order to detect the expected differences between the two groups of 0.2 units was 71 individuals.

The exclusion criteria were: (1) having a neurological disorder or a primary psychiatric disorder that could affect cognitive function such as intellectual disability, an organic mental disorder or an active psychotic disorder (assessed through semi-structured, face-to-face, clinical interview in the case of the experimental group and by direct questions in the case of the healthy controls), (2) having a learning disorder or a head injury with loss of consciousness for more than 2 min and (3) use of drugs or psychostimulants that could interfere with the evaluation or the treatment. Additionally, the exclusion criteria for the control group of healthy individuals were: (1) had an Axis I (DSM-5) mental disorder. No potential participants in either the experimental or control group were excluded on the basis of exclusion criteria 1, 2, or 3.

The Ethics Committee of CEIC Fundació Unió Catalana d’Hospitals (CEIC14/71) approved the study, and informed consent (signed document) was obtained from all the participants.

### Procedure

First, a face-to-face clinical interview and a functional analysis adapted from the semi-structured clinical interview SCID-I (39) to evaluate CSB, was performed by experienced psychologists (more than 7 years of clinical experience in behavioral addictions). The questions included in this interview include functional impairment (e.g., functional impairment in familial relationships, other social relationships, and academic achievement), preoccupation, withdrawal, loss of control, problematic and non-problematic sexual behaviors, escaping from adverse mental states, and questions regarding demographic data.

A second visit, with an average duration of 90 min, was scheduled within a week where participants completed the below-mentioned questionnaires.

### Instruments

#### Sexual Compulsivity Scale

This is a 10-item Likert-type psychometric scale that measures tendencies toward sexual preoccupation and hypersexuality (37). The Spanish version of the inventory has demonstrated satisfactory psychometric properties (Cronbach’s alpha coefficient 0.83 and 0.72) in men and women (40), and its divided in two sub-scales: interference of sexual behavior and failure to control sexual impulses.

#### Temperament and Character Inventory-Revised

This questionnaire has 240 items (41) with five-point Likert response options (42), and measures seven dimensions of personality: four of temperament (Harm Avoidance, Novelty Seeking, Reward Dependence, and Persistence) and three character dimensions (Self-Directedness, Cooperativeness, and Self-Transcendence). The scale has been translated and validated to Spanish, demonstrating satisfactory psychometric properties (43).

#### Symptom CheckList-90 Items-Revised

This Symptom CheckList-90 Items-Revised (SCL-90-R) (44) evaluates psychopathological symptoms and psychological problems. It is made up of ninety items and measures nine primary symptom dimensions: somatization, obsession-compulsion, interpersonal sensitivity, depression, anxiety, hostility, phobic anxiety, paranoid ideation, and psychoticism. The questionnaire also includes three global indices [global severity index (GSI), measuring overall psychological distress; positive symptom distress index (PSDI), to measure the intensity of symptoms; and positive symptom total (PST)]. The Spanish version of this scale has been validated (45), and presents a good internal consistency (mean Cronbach’s alpha = 0.75).

#### State-Trait Anxiety Index

This questionnaire (46) includes forty items on a 4-point rating scale and is self-reported, measuring state anxiety (20 items) and trait anxiety (20 items). The minimum score is 20 and the maximum is 80 points. The state anxiety uses items that measure subjective feelings of apprehension, tension, nervousness, worry, and activation/arousal of the autonomic nervous system and evaluates the current state of anxiety. The trait anxiety scale includes general states of calmness, confidence, and security and evaluates relatively stable aspects of “anxiety proneness.” The State-Trait Anxiety Index (STAI) has been translated to Spanish and validated in the Spanish population with a mean Cronbach’s alpha coefficient of 0.92 (47).

#### The Barratt Impulsiveness Scale

This is a 30-items scale rated on a four-point Likert scale (48) designed to measure the personality/behavior construct of impulsiveness and divided into three subscales including attentional key, motor key, and non-planning key, to determine overall impulsiveness score. It is translated and validated to Spanish (49).

#### Sociodemographical Variables

Additional demographic, clinical, and social/family variables related to sexual behaviors were measured using a semi-structured face-to-face clinical interview, including age, sex, duration of the problem, self-reported sexual orientation (heterosexual, homosexual, and bisexual) and education level (the reported education level was classified into the following variables: no studies, primary education studies, secondary education studies, and university studies).

#### Additional Clinical Variables

Other variables collected during the semi-structured face-to-face clinical interview were related with other clinical characteristics.

Participants were asked, “Have you been diagnosed with any physical illness in the last 12 months?” and “Do you currently take any medications regularly?” A physical illness was defined as a medical disorder that has been confirmed by an available mechanical, laboratory or imaging test, in contrast to a mental disorder diagnosed only by behavioral syndrome. The reported illnesses were classified into Sexual Transmission Diseases (defined as infections that are passed from one person to another through sexual contact), and no Sexual Transmission Diseases.

Participants were also asked about drug and alcohol regular consumption (defined as weekly or almost weekly consumption) in the past 12 months, the options included cannabis, cocaine, heroin, hallucinogens, and synthetic drugs. The reported drug uses were classified into two variables: Drug Use, and no Drug Use.

Finally, the following sexual problematic behaviors were evaluated: use of prostitution (defined as the practice of paying for engaging in sexual activity with someone), cruising (defined as wandering in an area picking up a sexual partner for anonymous sex), multiple sexual partners (defined as engaging in sexual activities with two or more new people within the last year), online pornography consumption (defined as access to sexually explicit content made available online in various formats including images and video files) and cybersex (defined as the use of the Internet to engage in sexually gratifying involving another person, through sex chats, sex webcams, or sexual direct messaging).

### Statistical Analysis

Differences between both experimental groups and between the clinical group as a whole and the control group were studied for each of the measures.

Standard graphical exploratory data analysis (mostly probability and quantile–quantile plots) was performed in numerical measures.

Numerical and categorical variables were used. We compared the means and the proportions among the clinical group as a whole and control group applying a Welch’s t-test and a Chi-squared test, respectively. Particularly, for numerical variables, we tested the normality assumption with a Shapiro–Wilk test. When the normality assumption was broken, a Mann–Whitney *U* test was performed to assess differences between groups. Particularly for categorical variables, we applied Fisher’s exact test when the number of expected observations was less than 5. For all test, Levene’s test were carried out to assess the assumption of equality of variances.

We applied a one-way ANOVA to assess differences among the two experimental and control groups. When homoscedasticity assumption was not accomplished according to Bartlett’s test, Welch’s ANOVA was computed. When the normality assumption was not accomplished, Kruskal–Wallis test was performed. We note that the results of these tests determined that there are significant statistical differences among the means of the three groups. Thus, we focused on the two-mean comparison tests in the reporting of the results. The Cohen’s *d* was computed to assess the effect size of both parametric and non-parametric pairwise comparisons between groups, in which the effect size | *d*| was considered low at values lower than 0.50, moderate between 0.50 and 0.80, and high at values greater than 0.80. Additionally and particularly in the non-parametric setting, the Cohen’s *d* effect size is calculated *via d* = 2*r*/*Z*/√(1−r2), which was proposed by Rosenthal (50) and where *r* = *Z*/√(*N*), *Z* is the *z*-score, and *N* is the sample size.

A two-sided *p*-value < 0.05 was considered statistically significant. All analyses were carried out using Python 3.8.8 (50).

## Results

### Sociodemographic Variables

The socio-demographic characteristics of the groups are represented in Table 1. In terms of problematic sexual behaviors, the CSB non-online group exhibited the following behaviors: 59% use of prostitution, 23% cruising, and 18% multiple sexual partners. None of the patients of this group were engaged in any type of problematic online sexual behavior. For the OCSB group, the problematic sexual behaviors were the following: 86% online pornography consumption and 14% cybersex.

**TABLE 1:** Socio-demographic variables of the experimental groups and control group.

	OCSB (*N* = 36)	Non-OCSB (*N* = 44)	CG (*N* = 25)
Age (years); mean (SD)	42.2 (10.0)	43.5 (11.9)	43.8 (16.7)
Employed, %	68.4	68.2	56
Education level, % *primary or less*	30.5	34.1	16
Living with a partner, %	75	56.8	56
Duration of the problem (years); mean (SD)	6.3 (7.4)	7.7 (8.0)	–
Tobacco use, %	27.8	29.5	20.1
Sexual orientation, % *heterosexual*	91.7	65.8	88
Sexual physical illness present %	20	46.5	4
Drug use, %	13.9	20.4	0.0
Alcohol use, %	2.8	6.8	0.0

*OCSB, online compulsive sexual behavior; non-OCSB, non-online compulsive sexual behavior; CG, control group; SD, standard deviation.*

The mean age for the online CSB, non-online CSB and Control group were 42.25 (±10.0), 43.5 (±11.9), and 43.8 (±16.7), respectively. Table 1 shows the comparison of those three groups according to sociodemographic measures. There were no statistically significant differences in most of the variables analyzed apart from physical illness present, where Sexual Transmission Diseases (HIV) were reported by some non-online CSB patients; and patient sexual orientation, where non-online CSB patients tended to have a higher percentage of homosexual (29.5%) and bisexual orientation (4.5%) than the other two groups.

### Clinical and Personality Characteristics of the Experimental and Controls Groups

#### Clinical Comparison

Table 2 gives the means and the results of the independent samples tests for all subscales to measure the differences in clinical variables between the experimental and control groups.

**TABLE 2:** Comparison between experimental and healthy control groups of psychopathological outcomes.

	CSB (*N* = 80)	CG (*N* = 25)				
			**Mann–Whitney test**			
	**Mean diff (SD)**	**Mean diff (SD)**	***U***		**Sig. (two-tailed) 95%**	**Cohen’s *d***

SCS						
Inference	13.6 (3.7)	5.4 (0.76)	26.5		<0.001	2.12
Control failure	14.0 (4.21)	5.6 (1.15)	12.5		<0.001	2.05
TOTAL SCS	27.3 (7.06)	11.0 (1.87)	40.5		<0.001	2.19
SLC-90-R						
Somatization	0.995 (0.76)	0.32 (0.31)	432.0		<0.001	0.92
Sensitivity	1.27 (0.92)	0.23 (0.25)	270.0		<0.001	1.27
Depression	1.55 (0.89)	0.32 (0.29)	181.0		<0.001	1.5
Anxiety	1.13 (0.83)	0.29 (0.28)	294.0		<0.001	1.21
Hostility	1.02 (0.92)	0.24 (0.37)	386.5		<0.001	1.01
Phobia	0.63 (0.78)	0.07 (0.14)	457.0		<0.001	0.87
Paranoia	1.12 (0.84)	0.15 (0.24)	271.5		<0.001	1.27
Psychoticism	1.24 (0.79)	0.14 (0.23)	148.5		<0.001	1.6
Overall severity	1.22 (0.7)	0.28 (0.2)	170.0		<0.001	1.54
STAI						
Anxiety state	24.0 (12.6)	10.2 (8.19)	319.5		<0.001	1.15

			**Independent sample tests**			
	**Mean diff (SD)**	**Mean diff (SD)**	***t***	**df**	**Sig. (two-tailed) 95%**	**Cohen’s *d***

SCL-90-R						
Obsession-compulsion	1.48 (0.78)	0.45 (0.34)	9.18	103	<0.001	1.7
STAI						
Anxiety trait	28.0 (11.1)	13 (6.16)	8.52	103	<0.001	1.67

*CSB, compulsive sexual behavior group; CG, control group; SD, standard deviation.*

Significant differences were observed in all of the psychopathology measures in the comparison between CSB patients and controls (Table 2). Taking into account the compulsive sex problem severity according to the Sexual Compulsivity Scale (SCS) questionnaire results, statistically significant differences can be observed for all variables (total compulsive behavior, interference of sexual behavior and failure to control sexual impulses) with high effect size (*d* > 1). Regarding the SLC-90-R questionnaire, CSB patients obtained higher scores than controls. Additionally, the effect size was large in all of the variables (*d* > 0.8). Even greater differences can be observed in the STAI test, where the score on anxiety (both trait and state) is higher among CSBs than among control with a large effect size (*d* > 1) in Anxiety trait.

#### Personality Comparison

Regarding personality traits (see Table 3), we observed significantly higher scores, with a moderate effect size, in harm avoidance and self-transcendence subscales among CSB patients when compared with controls. Additionally, there were also significant differences in self-directedness, cooperativeness and self-directness, where CSB patients showed lower scores than controls.

**TABLE 3:** Comparison between experimental and healthy control groups of personality outcomes.

	CSB (*N* = 80)	CG (*N* = 25)				
			**Independent sample tests**			
	**Mean diff (SD)**	**Mean diff (SD)**	***T***	**df**	**Sig. (two-tailed) 95%**	**Cohen’s *d***

TCI-R						
Novelty seeking	105.25 (15.97)	99.16 (13.6)	1.70	103	0.09	0.41
Harm avoidance	104.65 (21.4)	91.88 (15.6)	3.20	103	0.002*	0.68
Reward dependence	99.1 (14.2)	104.1 (13.1)	−1.54	103	0.127	−0.36
Persistence	107.3 (19.8)	106.4 (21.1)	0.20	103	0.842	0.05
Self-directedness	124.8 (23.1)	158.68 (17.4)	−6.70	103	<0.001*	0.63
BIS						
Motor	17.6 (7.4)	12.4 (7.21)	3.04	103	0.002*	0.71

			**Mann–Whitney test**			
			***U***		**Sig. (two-tailed) 95%**	**Cohen’s *d***

TCI-R						
Cooperativeness	131.44 (18.7)	141.36 (12.6)	639.5		<0.001*	0.55
Auto-transcendence	67.2 (15.2)	52.3 (11.3)	428.0		<0.001*	0.93
BIS						
Cognitive	16.7 (5.4)	12.2 (4.8)	482.0		<0.001*	0.82
Unplanned	20.4 (8.0)	15 (6.8)	578.0		0.001*	0.65

CSB, compulsive sexual behavior group; CG, control group; SD, standard deviation. ‘*’ means that the effect is considered statistically significant.

Regarding the Barratt Impulsiveness Scale (BIS), there were significant differences in their component variables: cognitive impulsivity, motor impulsivity, and unplanned impulsivity, which have a moderate to high effect size, where the clinical group scores higher in all the variables.

### Comparison Between Non-online Sexual Behaviors and Online Sexual Behavior Patients

Table 4 reports the results of the comparison between OCSB and non-OCSB patients on socio-demographic variables. There were no statistically significant differences in most of the variables analyzed apart from patient sexual orientation and sexual physical diseases present. The non-OCSB group tended to have a higher percentage of homosexual (29.5%) and bisexual orientation (4.5%) than the OCSB group, which was more commonly represented by heterosexual orientation (91.7%).

**TABLE 4:** Comparison between OCSB and non-OCSB patients of socio-demographic variables.

	OCSB (*N* = 36)	Non-OCSB (*N* = 44)				
			**Independent sample tests**			
			***t***	**df**	**Sig. (two-tailed) 95%**	**Cohen’s *d***

Age (years); mean (SD)	42.2 (10.0)	43.5 (11.9)	0.48	78	0.63	0.11

			**Chi-squared test**			
			**χ ^2^**	**df**		

Employed, %	69.4	68.2	0.01	2	0.9	–
Education level, % *primary or less*	30.5	34.1	1.06	3	0.79	–
Sexual orientation, % *heterosexual*	91.7	65.8	7.94	2	0.02*	–
Sexual physical diseases present, %	20	46.5	6.61	1	0.01*	–
Drug use, %	13.9	20.4	0.22	1	0.64	–
Alcohol use, %	2.8	6.8	0.10	1	0.76	–

			**Mann–Whitney test**			
			***U***		**Sig. (two-tailed) 95%**	**Cohen’s *d***

Duration of the problem (years); mean (SD)	6.27 (7.4)	7.67 (8.0)	717.0		0.23	0.16

OCSB, online compulsive sexual behavior; non-OCSB, non-online compulsive sexual behavior; SD, standard deviation. ‘*’ means that the effect is considered statistically significant.

Table 5 shows the comparison between OCSB and non-OCSB on psychopathology, as measured by the SCL-90-R and STAI personality characteristics, and problem severity as measured by the SCS questionnaire.

**TABLE 5:** Comparison between OCSB and non-OCSB patients of psychopathological outcomes.

	OCSB (*N* = 36)	Non-OCSB (*N* = 44)				
			**Independent sample tests**			
	**Mean diff (SD)**	**Mean diff (SD)**	***t***	**df**	**Sig. (two-tailed) 95%**	**Cohen’s *d***

SCS						
Inference	12.9 (3.26)	14.2 (3.88)	1.44	78	0.15	0.34
Control failure	12.8 (3.9)	15.1 (4.17)	2.47	78	0.01*	0.58
TOTAL SCS	25.7 (6.47)	28.8 (7.23)	1.89	78	0.06*	0.45
SLC-90-R						
Obsession-Compulsion	1.45 (0.78)	1.5 (0.8)	0.28	78	0.78	0.06
Paranoia	0.97 (0.76)	1.25 (0.88)	1.50	78	0.14	0.34
Psychoticism	1.07 (0.66)	1.38 (0.87)	1.77	78	0.08	0.4
Overall severity	1.11 (0.57)	1.32 (0.79)	1.39	78	0.17	0.31
STAI						
Anxiety state	22.9 (9.63)	24.9 (14.6)	0.7	78	0.48	0.15
Anxiety trait	26.3 (11.1)	29.4 (11.0)	1.23	78	0.22	0.28

			**Mann–Whitney test**			
			***U***		**Sig. (two-tailed) 95%**	**Cohen’s *d***

SCL-90-R						
Somatization	0.93 (0.62)	1.05 (0.85)	759.5		0.38	0.07
Sensitivity	1.16 (0.8)	1.37 (1.0)	702.5		0.19	0.20
Depression	1.48 (0.8)	1.62 (0.95)	737.5		0.30	0.12
Anxiety	0.9 (0.66)	1.33 (0.9)	576.5		0.02*	0.48
Hostility	0.78 (0.65)	1.21 (1.06)	626.0		0.05	0.36
Phobia	0.61 (0.74)	0.64 (0.8)	764.5		0.40	0.06

OCSB, online compulsive sexual behavior; non-OCSB, non-online compulsive sexual behavior; SD, standard deviation. ‘*’ means that the effect is considered statistically significant.

Significant differences were observed on the Anxiety subscale of the SCL-90-R, where non-OCSB obtained higher scores, with a moderate effect size (*d* = 0.48). With respect to problem severity, patients with non-OCSB obtained significantly higher scores at the SCS Impulse control failure scale. No significant differences were found in other measures.

Regarding the Temperament and Character Inventory-Revised (TCI-R) questionnaire, and BIS-11 questionnaire no statistically significant differences were found between OCSB and non-OCSB patients in any of the personality dimensions. The measure of the effect size on each variable is considered low apart from novelty seeking (*d* = 0.35), and motor impulsivity (*d* = 0.43), higher in non-OCSB, and self-directness (*d* = 0.48), lower in non-OCSB, which were near to be considered moderate.

## Discussion

The purpose of this study was to evaluate the clinical, personality and socio-demographic characteristics of patients diagnosed with CSB or with OCSB using a case-control design; and to compare both clinical groups on socio-demographics, sexual behavior, sexual problem severity, psychopathology and personality characteristics. A sample of 87 patients was recruited from a Behavioral Addictions unit at an urban mental health center in Barcelona.

As in previous studies (18), when compared to healthy controls, both experimental groups showed higher psychopathology, psychological problems in all measures and higher CSB. As observed in other studies (51), it may be that the reason for consultation is sexual behavior and, after psychopathological examination, a psychiatric pathology is detected. According to this, the presence of CSBs might be indicative of the need of an in-depth examination of other Axis-I pathologies that might otherwise go unnoticed.

With regard to personality, compared to healthy controls, patients scored higher in harm avoidance and self-transcendence, and lower in self-directness and cooperativeness. This is in line with what was found in the few articles analyzing the personality of this type of patient (52). High harm avoidance has been associated with affective disorders, anxiety disorders, and substance abuse (41). Moreover, these personality profile, combined with high impulsivity, is similar to those found in other behavioral addictions and substance addictions, where the results of many studies showed that patients with gambling disorder scored significantly higher on the temperament dimension harm avoidance; whereas they scored significantly lower on the character dimensions Self-Directedness and Cooperativeness (53–55). Even so, there are some differences with gambling disorder patients, for example, there is no difference with respect to controls on the novelty seeking scale. Other authors (52) have found a similar results in CSB subjects where openness to experience was not found to predict hypersexual behavior. This personality profile could indicate that compulsive sex could be more related to a coping strategy than to an intention to live new experiences, that these types of patients have little control regarding their sexual behaviors, and that they will continue to carry out these behaviors to in spite of the negative consequences that can be caused to them.

In respect of, self-transcendence, which measures the spiritual behavior of each individual (characteristics of spirituality, mysticism, magical, and religious thoughts) has been found to be higher in the clinical groups. This result is in line with other studies where the authors found higher religious believes in CSB population (56), and in contrast with authors founding a total absence of religious beliefs in similar samples (57). The restrictive morality of some religions with respect to sexual behavior could cause the individual to consider their sexual behavior out of control when compared with the “rules” of the religion that is lived. In this sense, some authors have suggest that emotions like guilt and shame may temporarily inhibit some sexual behaviors in hypersexual persons’, but those only act as inhibitors in a short-term due the increased sexual urge and the difficulties in the suppression of this desire (8). It would be of interest to study whether it is morality itself that facilitates this experience, or it is personality traits and their coping strategies that determine the way in which the person understands and incorporates their beliefs.

Finally, the clinical group scored higher in all impulsivity scales. Other authors have found links between CSB and self-report or task related measures of impulsiveness and impulsivity scores (8, 58, 59). Although some authors defend that the CSB is an impulse control disorder, and that the behavior in these patients may be a consequence of a deficit in the inhibitory control system, this type of impulsiveness has also been found in other behavioral additions, specifically in gambling disorder. Therefore, in the future it would be interesting to further explore the mediating relationship of impulsivity in the uncontrolled sexual behavior.

When comparing OCSB patients with non-OCSB, the differences were limited to non-OCSB exhibiting higher physical sexual diseases presence and higher percentage of homosexual and bisexual orientation. Moreover, non-OCSB obtained higher scores in anxiety and in sexual impulse control failure.

In the actual literature, few is known about the relationship between sexual orientation and sexual behaviors in CSB, with some authors finding lower levels of capability to control sexual urges and fantasies, and higher negative consequences of this behavior in gay, bisexual, transgender, and queer (LGTBQ) males (60). Easier access to casual sexual encounters (through cruising, saunas, sex parties…) would facilitate the appearance of the problem, as occurs in other behavioral addictions (e.g., gambling disorder), where it has been found that countries with easier access to this content has higher prevalence of problematic gambling (61).

Even so, considering our findings, the higher percentage of males consulting for any type of CSB were heterosexual. This result is in line with previous studies, suggesting that CSB is more prevalent among men than women, but differs from those authors finding in general population, more prevalence among LGBTQ men than heterosexual men (60, 62–64). It is possible that the stigmatization associated with homosexuality and that the more liberal sexual behaviors that are usually associated with this sexual orientation, lead these people to seek less treatment for this problem, consulting for other comorbid symptoms (i.e., anxiety).

Non-online compulsive sexual behavior patients showed more sexually transmitted diseases (STD). It is plausible that the effortless accessibility, high variety, and the large number of unknown sexual partners that implies the non-OCSB contribute to the uncontrollable engagement in risky sexual activities that lead these patients to develop STD.

Finally, our results have implications for clinical practice. First, both CSB (online and non-online) patients may experience a variety of comorbid psychological and medical problems. Second, the few differences between OCSB and non-OCSB personality traits suggest underlying common vulnerabilities and nosological similarities independently of the sexual behavior type. Even so, our results seem to indicate that people with non-OCSB may suffer more consequences that are negative, and that these may cause more severe comorbid symptoms, therefore, the clinical approach should have these repercussions into account. Whether sexual orientation plays a role in treatment response in CSB, and whether treatment needs differ based on sexual orientation, necessitates further inquiry.

Several methodological limitations to this study need to be taken into account. First, the results are based on a small sample. It is therefore difficult to generalize the conclusions. Second, the participants in the sample are only representative of people who seek treatment, and therefore the findings obtained may not apply to all individuals with CSB. Even so, these results may be of value for patients seeking treatment in relation to CSB. Third, the assessment trough standardized self-administered questionnaires did not allow for in-depth evaluation of specific Axis I and II comorbid disorders, although it should be noted that patients were supervised by a trained psychologist to ensure the highest quality of data collection. Fourth, the retrospective design to determine some sexual behaviors might be confounded by memory biases of the patients.

## Data Availability Statement

The raw data supporting the conclusions of this article will be made available by the authors, without undue reservation.

## Ethics Statement

The studies involving human participants were reviewed and approved by the Ethics Committee of CEIC Fundació Unió Catalana d’Hospitals (CEIC14/71). The patients/participants provided their written informed consent to participate in this study.

## Author Contributions

DF, VG-B, and JS conceived and planned this manuscript. VG-B and JS carried out the search and revision of the literature, and drafted the study. DF and OC-P analyzed the data. VG-B, JS, OC-P, DF, MB-A, SJ-M, AH, AP-G, and JR revised the manuscript critically for important intellectual content, and commented on and approved the final manuscript and were accountable for all aspects of the work. All authors have read and agreed to the published version of the manuscript.

## Conflict of Interest

The authors declare that the research was conducted in the absence of any commercial or financial relationships that could be construed as a potential conflict of interest.

## Publisher’s Note

All claims expressed in this article are solely those of the authors and do not necessarily represent those of their affiliated organizations, or those of the publisher, the editors and the reviewers. Any product that may be evaluated in this article, or claim that may be made by its manufacturer, is not guaranteed or endorsed by the publisher.
